# Recurrent syncope due to ischemia with non-obstructive coronary artery disease: a case report

**DOI:** 10.1186/s13256-023-04316-y

**Published:** 2024-03-12

**Authors:** Bihan Huang, Xueying Han, Peiyi Xie, Shaoyuan Chen

**Affiliations:** 1 Department of Cardiology, Huazhong University of Science and Technology Union Shenzhen Hospital, Shenzhen, China; 2 Department of Intensive Care, Huazhong University of Science and Technology Union Shenzhen Hospital, Shenzhen, China; 3 Department of Cardiology, The 6th Affiliated Hospital of Shenzhen University Medical School, Shenzhen, China

**Keywords:** Ischemia and no obstructive coronary artery disease (INOCA), Coronary artery spasm (CAS), Coronary microvascular dysfunction (CMD), Case report

## Abstract

**Background:**

Ischemia with non-obstructive coronary artery disease is a prevalent form of ischemic heart disease. The majority of ischemia with non-obstructive coronary artery disease cases are attributed to underlying factors such as coronary microvascular dysfunction (CMD) and/or coronary artery spasm. Ischemia with non-obstructive coronary artery disease can present with various clinical manifestations. Recurrent syncope is an atypical complaint in patients with ischemia with non-obstructive coronary artery disease.

**Case presentation:**

This case report describes the presentation of a 58-year-old Chinese male patient who experienced repeated episodes of syncope. The syncope was found to be caused by concomitant coronary artery spasm and presumptive coronary microvascular dysfunctionc suggested by “slow flow” on coronary angiography. The patient was prescribed diltiazem sustained-release capsules, nicorandil, and atorvastatin. During the three-month follow-up conducted on our outpatient basis, the patient did not experience a recurrence of syncope.

**Conclusion:**

This study highlights the importance of considering ischemia with non-obstructive coronary artery disease as a potential cause of syncope in the differential diagnosis. It emphasizes the need for early diagnosis of ischemia with non-obstructive coronary artery disease to facilitate more effective management strategies.

**Supplementary Information:**

The online version contains supplementary material available at 10.1186/s13256-023-04316-y.

## Introduction

Ischemia with no obstructive coronary arteries (INOCA) is a prevalent form of ischemic heart disease.

Around half of the patients who experience chest pain and with objective evidence of myocardial ischemia have are diagnosed with non-obstructive coronary artery disease (CAD) during angiography. Non-obstructive coronary artery disease is characterized by less than 50% narrowing in any major blood vessel surrounding the heart [1–3]. Coronary microvascular dysfunction (CMD) and vasospasm of the epicardial arteries are the two predominant etiologies of INOCA [4]. INOCA can present with various clinical manifestations, encompassing not only angina but also acute coronary syndrome, hospitalization for heart failure, and sudden cardiac death accompanied by life-threatening arrhythmias [5]. This study presents a case of a 58-year-old male patient who experienced repeated episodes of syncope as a result of concurrent coronary artery spasm (CAS) and presumptive CMD. This report was written according to the case report (CARE) guidelines [6]. We present our case report in accordance with the CARE checklist (Additional file 1).

## Case presentation

A male Chinese patient, aged 58 years, visited the cardiology clinic with recurring episodes of syncope for 20 days. He experienced episodes of transient loss of consciousness, each lasting approximately 30 seconds, accompanied by chest pain. The most recent episode of syncope occurred in the early morning and was characterized by dizziness and a temporary loss of consciousness. He had a history of hypertension.

He smoked one pack of cigarettes daily for about 20 years with frequent alcohol intake. During the examination of the patient’s cardiac system, auscultation indicated normal heart sounds and no murmurs. Magnetic resonance imaging (MRI) and magnetic resonance angiography (MRA) of the brain, laboratory tests measuring cardiac enzymes, and electrocardiogram (ECG) were normal.

**Fig. 1:**
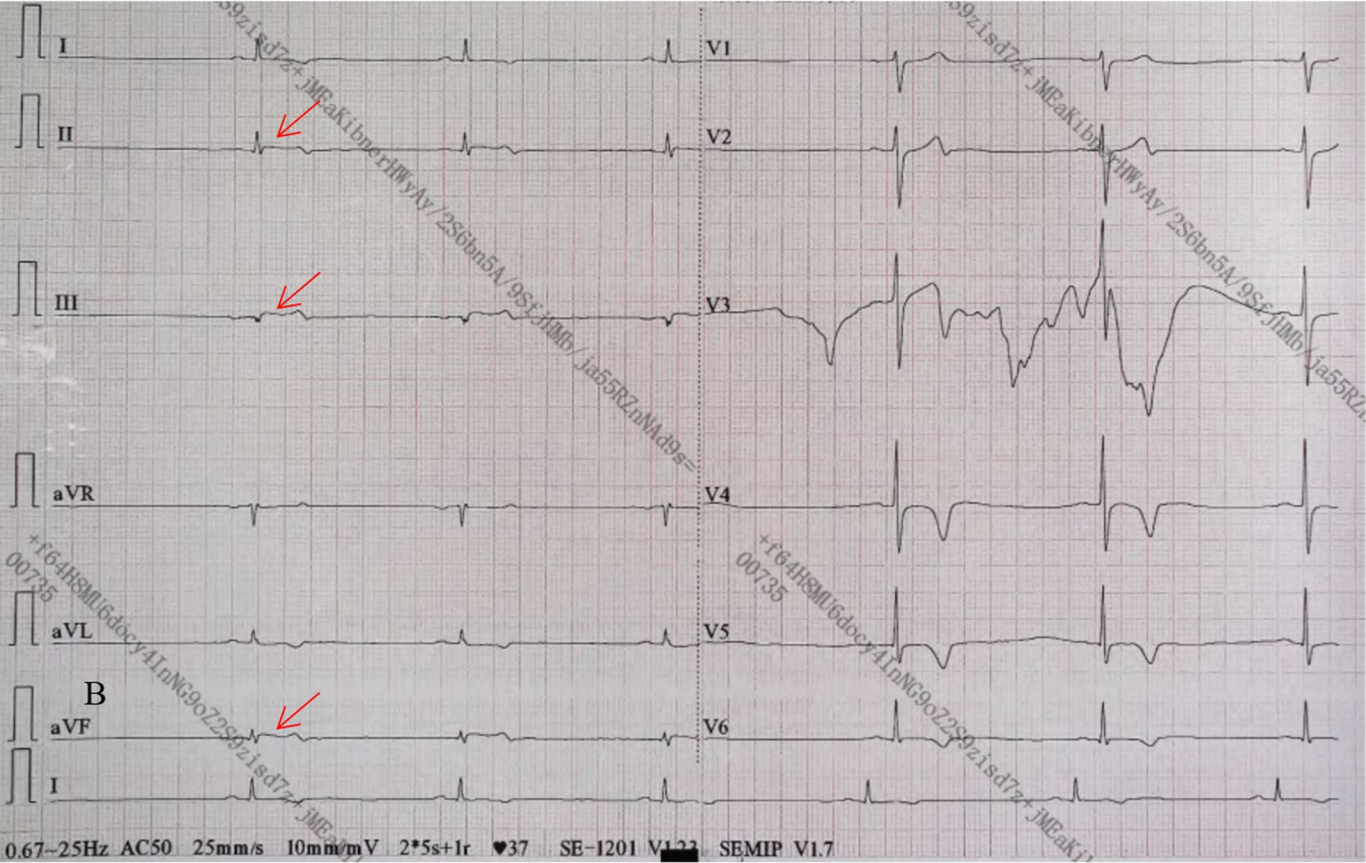
ECG: The arrows showed a 0.1 mv ST elevation at leads DII, III, and aVF

**Fig. 2:**
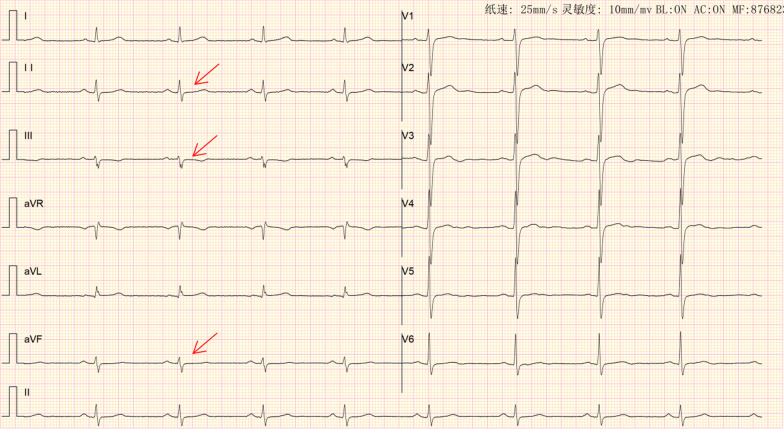
ECG: The arrows revealed normal sinus rhythm with no significant ST changes in all leads

**Fig. 3:**
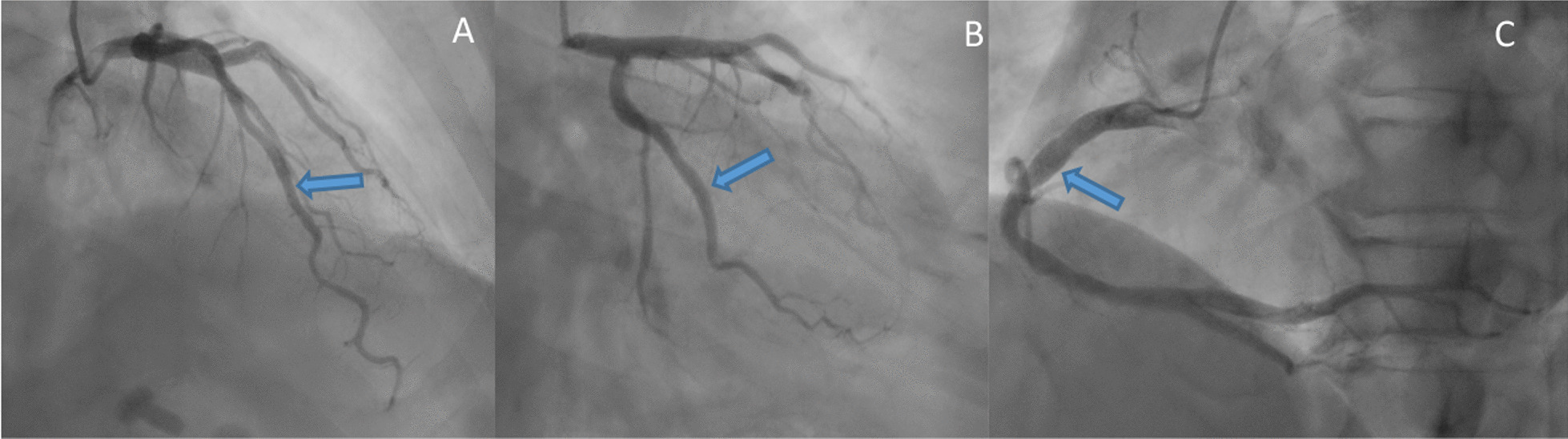
Coronary angiography: The arrows revealed nonobstructive coronary arteries in the left anterior descending artery (LAD; **A**), left circumflex artery (LCX; **B**), and right coronary artery (RCA; **C**)

A transthoracic echocardiogram provided confirmation of the left ventricle’s size and function being within normal parameters. Approximately 16 hours after admission, about at 5 a.m., during sleeping, the patient experienced a recurrence of sudden chest pain, diaphoresis, and subsequent syncope. Physical examination indicated a heart rate of 37 beats per minute (bpm), respiratory rate of 25 breaths per minute, and blood pressure of 62/38 mmHg. The ECG revealed dynamic ST-segment elevation of 0.1 mV in leads II, III, and augmented vector foot (aVF; Fig. 1). Syndromes spontaneously improved within a few minutes, the ECG returned to baseline and blood pressure improved to normal later (Fig. 2). Cardiac troponin I enzyme levels were negative. Coronary angiography (CAG) was not performed immediately due to the symptom relief. However, following the doctor’s advice, he had CAG examination 4 days later. Subsequent CAG confirmed diffuse coronary slow flow (thrombolysis in myocardial infarction flow grade 2), without any substantial flow-limiting lesions (Fig. 3, Additional files 2, 3, 4: Videos S1, S2, and S3). The patient did not agree to undergo coronary vasospasm provocation testing due to possible adverse event risk, and refused further examination to further confirm the cause due to financial reasons. However, based on the patient’s symptoms, ECG findings, and coronary angiography, a diagnosis of CAS and presumptive CMD was established. The patient was prescribed diltiazem sustained-release capsules (30 mg, four times daily), nicorandil (5 mg, three times daily), and atorvastatin (20 mg/day).

During the 3-month follow-up conducted on our outpatient basis, the patient successfully ceased smoking and effectively controlled his hypertension. Furthermore, he did not experience a recurrence of the previously reported chest discomfort, syncope, or any other symptoms.

## Discussion

A significant number of patients who had suspected CAD indicate normal or nonobstructive coronary arteries during invasive coronary angiography procedure [7]. Despite the fact that a significant number of these patients are categorized as having normal coronary arteries, the presence of ischemia without obstructive CAD has been linked to elevated cardiovascular risk and a greater likelihood of undergoing repeat coronary angiography [2, 8, 9]. The initial diagnosis of INOCA necessitates the identification of ischemic signs or symptoms, supported by objective evidence of myocardial ischemia through electrocardiography (ECG) or cardiac imaging. Additionally, the absence of flow-limiting obstruction as determined by coronary angiography is required [1].

There are two diagnoses being used interchangeably to denote the existence of angina pectoris in the absence of obstructive epicardial coronary stenosis: angina pectoris and no obstructive coronary artery disease (ANOCA) and INOCA. The INOCA is widely applied, including in expert consensus documents [10]. Many patients suffering from angina and lacking obstructive epicardial coronary stenosis may not present with signs of ischemia and findings of ischemia. ANOCA represents the broader and heterogeneous patient population. However, our patient experienced a sudden chest pain, and the ECG revealed dynamic ST-segment elevation. This case meets the diagnostic criteria of INOCA.

Coronary vascular dysfunction appears to be the primary factor contributing to ischemia in a significant proportion, ranging from 59% to 89%, of patients diagnosed with INOCA [11, 12]. This dysfunction encompasses both CMD and CAS [13, 14]. CMD may be characterized as a condition where the small blood vessels exhibit increased sensitivity to vasoconstrictor stimuli and reduced capacity for microvascular vasodilation [15].

Traditional risk factors for cardiovascular disease, such as dyslipidemia and hypertension, are known to contribute to the development of coronary microvascular dysfunction [16]. Systemic inflammatory disease also plays an important role in CMD [17]. Coronary vasospasm occurs as a result of dynamic obstruction of the epicardial coronary arteries due to a vasomotor disorder. Epicardial vessel spasm commonly occurs when a hyper-responsive segment of the epicardial coronary vessel contracts to its maximum extent following exposure to vasoconstrictor stimuli. These stimuli may include smoking, elevated blood pressure, or emotional stress. CAS occurrence has the potential to cause significant myocardial ischemia, acute myocardial infarction, or even sudden cardiac death [18]. Concomitant CAS and CMD are linked to a more unfavorable prognosis compared with the presence of either condition alone [19]. Our patient had underlying predisposition for his presentation with INOCA due to a preexisting medical history of hypertension and smoking.

The primary treatment approach, especially a calcium channel blockade such as diltiazem, is preferred as the first-choice treatment, as this drug has been shown to reduce microvascular constriction and alleviate spasms and is effective in managing patients with both coronary microvascular dysfunction and coronary spasm [20]. Nicorandil is also the recommended drug along with the first-line treatment. Nicorandil can reduce coronary microcirculatory resistance and expand coronary blood flow by activating adenosine triphosphate (ATP)-sensitive potassium (K_ATP_) channels on the vascular smooth muscle cell membrane [21].

## Conclusion

INOCA is associated with elevated cardiovascular risk. In our case, the patient exhibited repeated episodes of syncope caused by INOCA. In this population, it is imperative to strictly adhere to medical treatment and actively avoid risk factors. This study highlights the importance of considering INOCA as a potential cause of syncope in the differential diagnosis. It emphasizes the need for early diagnosis of INOCA to facilitate more effective management strategies.

### Supplementary Information

**Additional file 1: **CARE checklist.

**Additional file 2: Video S1. **Coronary slow flow in the LAD.

**Additional file 3: Video S2. **Coronary slow flow in the LCX.

**Additional file 4: Video S3. **Coronary slow flow in the RCA.

## Data Availability

The article includes the study’s original contributions, additional inquiries can be addressed to the corresponding author.
